# Characterization of introgression from the teosinte *Zea mays* ssp. *mexicana* to Mexican highland maize

**DOI:** 10.7717/peerj.6815

**Published:** 2019-05-03

**Authors:** Eric Gonzalez-Segovia, Sergio Pérez-Limon, G. Carolina Cíntora-Martínez, Alejandro Guerrero-Zavala, Garrett M. Janzen, Matthew B. Hufford, Jeffrey Ross-Ibarra, Ruairidh J. H. Sawers

**Affiliations:** 1 Unidad de Genómica Avanzada (LANGEBIO), Centro de Investigación y de Estudios Avanzados del Instituto Politécnico Nacional, Irapuato, Guanajuato, Mexico; 4 Department of Ecology, Evolution, and Organismal Biology, Iowa State University, Ames, IA, USA; 5 Department of Plant Sciences, Center for Population Biology, and Genome Center, University of California, Davis, CA, USA

**Keywords:** Maize, Teosinte, Introgression, Local adaptation

## Abstract

**Background:**

The spread of maize cultivation to the highlands of central Mexico was accompanied by substantial introgression from the endemic wild teosinte *Zea mays* ssp. *mexicana*, prompting the hypothesis that the transfer of beneficial variation facilitated local adaptation.

**Methods:**

We used whole-genome sequence data to map regions of *Zea mays* ssp. *mexicana* introgression in three Mexican highland maize individuals. We generated a genetic linkage map and performed Quantitative Trait Locus mapping in an F_2_ population derived from a cross between lowland and highland maize individuals.

**Results:**

Introgression regions ranged in size from several hundred base pairs to Megabase-scale events. Gene density within introgression regions was comparable to the genome as a whole, and over 1,000 annotated genes were located within introgression events. Quantitative Trait Locus mapping identified a small number of loci linked to traits characteristic of Mexican highland maize.

**Discussion:**

Although there was no strong evidence to associate quantitative trait loci with regions of introgression, we nonetheless identified many Mexican highland alleles of introgressed origin that carry potentially functional sequence variants. The impact of introgression on stress tolerance and yield in the highland environment remains to be fully characterized.

## Introduction

Maize (*Zea mays* ssp. *mays*) was domesticated in southwestern Mexico approximately 9,000 years ago from an annual wild grass, the teosinte *Zea mays* ssp. *parviglumis* (Matsuoka et al., 2002; Piperno et al., 2009). Following domestication, maize dispersed across Mexico and diversified to give rise to locally-adapted landrace varieties (Wellhausen et al., 1952; Sanchez & Goodman, 1992; Ruiz Corral et al., 2008; Perales & Golicher, 2014). Cultivated maize spread rapidly beyond the ancestral niche occupied by *parviglumis* (Piperno, 2006; Merrill et al., 2009; Grobman et al., 2012), raising questions as to the origin and nature of the genetic variation underlying adaptive phenotypic change. Within Mexico, the colonization of the highland Central Plateau represents a clear example of niche expansion with respect to the *parviglumis* ancestor (Piperno, 2006). The Mexican highlands present a unique combination of environmental challenges to maize cultivation: low temperatures necessitate planting early in the year in order that plants might reach maturity, in turn risking exposing seedlings to frost and water deficit before the onset of annual rains; throughout the growing season, low-temperature, high-levels of UV radiation and hail storms pose further difficulties (Eagles & Lothrop, 1994; Lafitte & Edmeades, 1997; Jiang et al., 1999; Mercer, Martínez-Vásquez & Perales, 2008); the volcanic origin of the central highland region, and the associated acidic nature of the soils, restricts the bioavailability of phosphorus (Bayuelo-Jiménez & Ochoa-Cadavid, 2014).

The first maize to be cultivated in the Mexican highlands encountered not only new environmental challenges, but also the highland-adapted teosinte *Zea mays* ssp. *mexicana* (hereafter, *mexicana*; Hufford et al., 2012a). Mexican highland maize and *mexicana* share morphological traits (most obviously, pronounced stem pigmentation and pubescence) that are rarely seen in *parviglumis* and lowland maize (Wellhausen et al., 1952; Wilkes, 1972). This common morphology presented the first indication of introgression between *mexicana* and maize, interpreted variously as the adoption of adaptive traits by maize or as mimicry by teosinte to evade removal from cultivated fields (Wilkes, 1972; Lauter et al., 2004). Subsequent molecular studies have demonstrated shared ancestry between Mexican highland maize and *mexicana* (Doebley, 1990; Matsuoka et al., 2002; Van Heerwaarden et al., 2011; Hufford et al., 2013). Introgression from *mexicana* to maize is more common than in the opposite direction, with *mexicana* contributing around 20% of the genome of Mexican highland maize varieties (Van Heerwaarden et al., 2011; Hufford et al., 2013; Romero Navarro et al., 2017; Wang et al., 2017). The use of increasing numbers of molecular markers and whole genome sequence datasets has allowed the mapping of large-scale regions of introgression within Mexican highland maize genomes (Hufford et al., 2013; Wang et al., 2017). A number of introgressed haplotypes are found at high frequency in Mexican highland maize, while being rare or absent in lowland populations, consistent with a possible role in local adaptation (Hufford et al., 2013; Takuno et al., 2015; Romero Navarro et al., 2017; Wang et al., 2017).

Introgression has been proposed as a mechanism whereby invasive populations can rapidly acquire the genetic variation necessary to adapt to local environmental challenges (Hedrick, 2013; Martin & Jiggins, 2017). For example, introgression of genes from *Heliconius melpomene* butterflies to *Heliconius timareta* has been linked to the acquisition of mimetic red wing patterns (Pardo-Diaz et al., 2012). Similarly, it has been suggested that the adaptation of *Arabidopsis arenosa* to serpentine soils was facilitated by introgression from the related species *A*. *lyrata* (Arnold et al., 2016). In cultivated barley, introgression from wild relatives appears to have aided expansion and adaptation (Poets et al., 2015). The expansion of weedy *Helianthus annuus* into certain regions of Texas was driven by admixture with the wild relative *Helianthus debilis*, promoting increased herbivore resistance (Whitney, Randell & Rieseberg, 2006; Whitney et al., 2015). Introgression from crops to their wild-relatives has also been documented, directly enhancing vigor (Gutierrez, Cantamutto & Poverene, 2011), or promoting persistence of weedy forms in cultivated fields through mimicry (Chen et al., 2004; Xia et al., 2011).

The fate of any given locus following hybridization will depend not only on the adaptive value of its associated variants but also the local genomic landscape in terms of recombination rates (RR) and the nature of linked genes (Martin & Jiggins, 2017). For example, studies in mice and humans have found weaker signals of introgression in areas of the genome where gene density is high and/or the RR is low (Sankararaman et al., 2014, 2016; Janoušek et al., 2015; Martin & Jiggins, 2017). In the context of crop plants, the potential benefits of introgression from wild relatives with respect to local adaptation or stress tolerance may be offset by decreased agronomic value as a result of linked pre-domestication gene content. It has been hypothesized that introgression will be facilitated by high local levels of recombination, allowing beneficial or neutral alleles to readily recombine away from undesirable variants (Barton & Bengtsson, 1986; Martin & Jiggins, 2017). Nonetheless, previous reports have described a number of Megabase-scale introgression events that have been transferred from *mexicana* to maize, associated with regions of low recombination in pericentromeric regions or chromosomal inversions (Hufford et al., 2013; Wang et al., 2017). If such large scale events are to be maintained by positive selection, the net effect of many hundreds of wild alleles must be beneficial. Although a low rate of recombination makes it hard to break linkage-drag, the flipside may be to promote introgression by holding together groups of beneficial alleles that collectively constitute an adaptive haplotype (Kirkpatrick & Barton, 2006; Twyford & Friedman, 2015; Marques, 2017).

In this study, we performed a detailed characterization of *mexicana* introgression in three Mexican highland maize individuals. Introgression events were characterized with respect to their size, gene content and variation. In addition, we generated a linkage map to estimate local RR. To assess the impact of gene flow on plant morphology and phenology, we mapped QTL for a number of traits in an F_2_ mapping population and compared their location to the introgression map.

## Materials and Methods

### Plant material

Sequence data reported here was generated from two outbred individuals of accession Mexi5 of the landrace Palomero Toluqueño (PT; collected near the city of Toluca, Mexico state at 19.29N, −99.57W, 2,597 masl), obtained from the collection of the International Center for Maize and Wheat Improvement (CIMMyT) seed bank, and one outbred individual of accession TC313 of landrace Mushito de Michoacán (MM; collected south of Pátzcuaro, Michoacán at 19.31 N, −101.68 W, 2,271 masl), from the collection of Alfredo Carrera, Universidad Autónoma Chapingo, Michoacán. Samples for sequencing were collected from mature field grown plants RS16-1032.6 (PT1), RS16-1032.9 (PT2) and RS13-1261.1 (MM). Individual PT1 was crossed as male to a partially inbred stock derived from the Mexican landrace Reventador (RV; an S6 derivative of accession Nay15, INIFAP; Bukowski et al., 2018), and a single individual of the resulting F_1_ stock self-pollinated to generate an F_2_ family. A total of 170 RV × PT1 F_2_ individuals were evaluated in a lowland field site (Valle de Banderas, Nayarit, Winter cycle 2017) and genotyped to generate a genetic linkage map and perform QTL mapping (see below).

### Whole genome sequencing

Total genomic DNA was extracted by LANGEBIO-CINVESTAV Genomic Services (http://www.langebio.cinvestav.mx/?pag=458). Whole genome sequencing was performed by NGX-Bio (San Francisco, California, USA) using the Illumina HiSeq 3000/4000 platform, using HiSeq 4000 SBS chemistry to generate 150 bp paired-end reads. A total of 85.5, 87.7 and 144.7 Gb of sequence was generated for PT1, PT2 and MM1, respectively. Genome sequence data is available from the National Center for Biotechnology Information (NCBI) Sequence Read Archive (SRA) database (PRJNA511379).

### Public sequence data

Additional whole genome sequence data for the lowland maize landraces Nal Tel (RIMMA0703) and Zapalote Chico (RIMMA0733) was taken from Wang et al. (2017) (SRP065483). An additional lowland maize sequence (BKN022), *mexicana* (TIL08 and TIL25), *Tripsacum* (TDD39103) and parviglumis (TIL01, TIL05, TIL10) were taken from Bukowski et al. (2018) (/iplant/home/shared/panzea/hapmap3/bam). Genome sequence was obtained in bam format, and aligned to the B73 reference genome v3 using BWA mem (BWA v.0.7.12; Li, 2013).

### Pre-processing of whole genome sequence data

The PT1, PT2 and MM1 sequence was processed using Trimmomatic v.0.32 (Bolger, Lohse & Usadel, 2014), set for pair-end data with the following parameters: LEADING: 3 TRAILING: 3 SLIDINGWINDOW: 4:15 MINLEN: 36. The resulting trimmed sequences, both paired and single end, were mapped against the B73 reference genome v3 using BWA v.0.7.12 (Li, 2013), under the default settings, using the -M option for Picard compatibility. The resulting sam output was sorted, and converted to bam format using Picard tools v.2.4.1 (http://broadinstitute.github.io/picard). Single-end and pair-end ordered bam files for each individual were merged using samtools v.1.3.1 (Li et al., 2009), and duplicated molecules removed with Picard tools using default parameters, with the flag REMOVE_DUPLICATES=true. The files were indexed using Picard Tools and indel realignment carried out with the genome analysis toolkit v.3.5.0 (McKenna et al., 2010). Publicly available lowland maize, *mexicana* and *Tripsacum* bam format sequences were processed using the same pipeline from the removal of duplicate molecules onward.

### Calculation of *D* and *f_d_*

Genotype likelihoods (GL) were calculated using ANGSD v.0.912 (Korneliussen, Albrechtsen & Nielsen, 2014) with the following parameters: -GL 1 -remove_bads 1 -nThreads 8 -doGlf 3 -doMajorMinor 1 -doMaf 1 -SNP_pval 1e-6 -minInd 2 -minMapQ 30 -minQ 20. Inbreeding values were calculated for each individual with ngsF v1.2.0 (Vieira et al., 2013), using the script ngsF.sh with the following parameters: –n_threads 20 –n_ind 9 –min_epsilon 1e-6 –glf <GL calculated with ANGSD> –n_sites <number of called SNPs>, giving the following values: RIMMA0703, 0.22; RIMMA0733, 0.30; TIL25, 0.59; TIL08, 0.66; TDD39103, 0.64: BKN022, 0.69: PT1, 0.00084; PT2, 0.00156; MM, 0.00042; TIL10, 0.49; TIL05, 0.56; TIL01, 0.48. Inbreeding coefficients were used in ANGSD SNP calling to account for deviations of the HWE using the following parameters: -SNP_pval 1e-6 -GL 1 -doMajorMinor 1 -doMaf 1 -rf -remove_bads 1 -minMapQ 30 -minQ 20 -minInd 4 -doGeno 4 -doPost 1 -postCutoff 0.95 -indF <inbreedingValues>. Allele frequencies were used to calculate *f*_*d*_ and D using the script ABBA_BABA.v1.pl (Owens, Baute & Rieseberg, 2016), based on the tree (((P1, P2), P3), O), where the P1 position was the three lowland genomes BKN022, RIMMA0703 and RIMMA0733, P2 was the three highland genomes PT1, PT2 and MM, P3 was the two teosinte *mexicana* genomes TIL08 and TIL25, and O was the *Tripsacum* genome TDD39103. From this analysis, we calculated the average value of *D* across the genome, along with an associated *p*-value estimated from the distribution of *D* values calculated in non-overlapping windows of five Mb across the genome (ABBA_out_blocker.pl; Jacknife_ABBA_pipe.R; ABBA_pvalue.R. Owens, Baute & Rieseberg, 2016). To calculate *D* at the level of individual chromosomes, we used non-overlapping windows of one Mb. To map introgression within the genome, a custom *R* script was used to calculate *f*_*d*_ in non-overlapping windows of 50 informative (ABBA/BABA) sites, based on the ABBA_BABA.v1.pl output. We considered the sets of the top 1% and 10% scoring windows as positive for introgression (Tables S1 and S2). Raw *f*_*d*_ output is provided in Table S3. We also generated a null data set, substituting South American for Mexican maize (Table S4). The average *f*_*d*_ across the whole genome was taken as the proportion of introgression. The location of gene models in introgression regions is listed in Table S5.

### Genotypic analysis of a RV × PT F_2_ family and construction of genetic linkage map

Total genomic DNA was extracted from 170 F_2_ individuals derived from the cross of RV × PT1 using the Qiagen (GmBH) DNeasy Plant Mini kit DNA extraction kit, according to the manufacturer’s instructions. Samples were analyzed by the International center for maize and wheat improvement (CIMMyT) using the DaRT platform (http://seedsofdiscovery.org/es/catalogo/saga-servicio-de-analisis-genetico-para-la-agricultura/; Sansaloni et al., 2011). Using tag sequences of ∼65 bp, a total of 26,727 single nucleotide polymorphisms (SNPs) were identified, with <50% missing data. Tags were anchored to physical positions in the B73 v3 reference genome using BLAST (Altschul et al., 1990), under the following parameters: min % for each base = 3, max % for each base = 60, *e*-value = 5*e*^−10^, max hits per sequence = 10, percent overlap = 90, percent identity = 90. Tags aligning to multiple positions and those that contained multiple SNPs, were discarded, as were tags derived from heterozygous sites for which only one allele could be aligned under the defined parameters, and sets of two or more tags that aligned to a common position. The exact position for each SNP was calculated on the basis of the position of the SNP within the tag and the position of the alignment of the tag against B73. The resulting set of 10,323 SNPs were transformed to hapmap format, and filtered to identify segregating sites (allele frequency >0.2 and <0.8), thinned to minimum spacing of one kb, and transformed to ABH format (A: PT; B: RV) using TASSEL v5.0 (Bradbury et al., 2007). The data were inspected visually using ABHgenotypeR v1.0.1 (Furuta et al., 2017), and passed to the ABHgenotypeR pipeline to impute missing data, corrected for under-called heterozygous sites, and corrected for single interspersed alleles using a maximum haplotype length of six. The proportion of missing sites dropped from 0.57 to 0.02 following imputation. The final proportion of sites was 24.4% A, 23.6% B, 52.0% H. Markers were assigned to linkage groups and ordered based on the B73 v3 physical map prior to estimation of the genetic map using R v3.4.0 (R Core Team, 2014) with R/QTL v1.41.6 (Broman et al., 2003), following the recommendations available at http://www.rqtl.org/tutorials/geneticmaps.pdf. The marker set was reduced once more on the basis of redundancy in the genetic map using the functions qtl::findDupMarkers and qtl::drop.markers, resulting in a final set of 1,166 SNPs that was passed to the function qtl::est.map with the kosambi mapping function. Genetic and physical distances were extracted per chromosome using qtl::pull.map. Local estimates of RR (cM/Mb) were obtained using R/MareyMap v1.3.4 (Rezvoy et al., 2007), fitting a cubic spline across each chromosome, using the parameters spar = 0.05 and d*f* = 10 (Tables S6 and S7). Markers that distorted the monotonic increase of the fitted spline were removed by hand to avoid negative rates. The genetic map of the maize nested association mapping population was obtained from MaizeGDB (Andorf et al., 2016), and physical positions were taken as the midpoint of genes associated with the markers, with local estimates of RR calculated as described above.

### Functional annotation of sequence variants

The SNPs obtained with ANGSD were converted to hapmap format using custom scripts, passed to TASSEL v5.2.43, and converted to vcf format, the reference allele at any given site being defined based on the B73 reference genome v3, set using bcftools v1.5. To perform functional annotation, the vcf file was passed to SnpEff v4.3 (Cingolani et al., 2012; Table S8). A custom *R* script was used to select SNPs homozygous for the alternative allele in the three highland maize genomes. Population differentiation data for Mexican highland and lowland maize populations was taken from Takuno et al. (2015). Maize gene annotation was taken from maize-GAMER (https://dill-picl.org/projects/gomap/maize-gamer/).

### QTL mapping of variation in morphological traits and flowering time

The 170 F_2_ RV × PT1 individuals used for the linkage mapping were grown to maturity in a lowland winter nursery (Valle de Banderas, Nayarit, Mexico, 20.8 N, −105.2 W, 54 masl), and evaluated for the following traits: plant height (PH), ear height (EH), stem pigment intensity (INT), stem pigment extent (EXT), stem macrohair pattern (MPAT); stem macrohair density (MDEN), tassel (male inflorescence) branch number (TBN), tassel length (TL) and days-to-anthesis (DTA). INT was evaluated on a semi-quantitative scale from zero to four. EXT was scored as 0%, 25%, 50%, 75% or 100%. MAPT was scored as 0 (no stem macrohairs), 1 (marginal macrohairs only), 2 (patchy macrohair production on the sheath) or 3 (uniform macrohair production on the sheath). MDEN was scored semi-quantitatively from zero to four. Other traits were evaluated as described previously (Flint-Garcia et al., 2005). QTL mapping was conducted using a single-scan in R/QTL (Broman et al., 2003), with the support of R/QTLtools (Lovell et al., 2018). Phenotypic data and the genetic map are provided as an R/QTL cross object in Table S9.

## Results

### Introgression from *mexicana* is distributed throughout the genome of Mexican highland maize

To characterize introgression from *mexicana* to Mexican highland maize, we generated whole genome sequence data from two outbred individuals of the landrace Palomero Toluqueño (PT1 and PT2) and a single outbred individual of the landrace Mushito de Michoacán (MM1), yielding a coverage of ∼40 fold for PT1 and PT2, and ∼70 fold for MM1 (Table S10). In total, we identified 71,623,944 SNPs across the three individuals. To estimate the extent of *mexicana* introgression, we calculated Patterson’s *D* statistic (Durand et al., 2011) and genome-wide *f*_*d*_ (Martin, Davey & Jiggins, 2015). Briefly, working with genomic sequence from highland and lowland maize, *mexicana* and the related grass *Tripsacum* (see Materials and Methods), we identified those sites that were polymorphic between *Tripsacum* and *mexicana*, and compared the frequency with which highland maize carried the *mexicana* allele and lowland maize the *Tripsacum* allele (the “ABBA” pattern) to the frequency of the complementary case (the “BABA” pattern) (Green et al., 2010). A total of 905,537 SNPs were characterized as following either the ABBA or BABA pattern, and, therefore, to be informative for the analysis. Our analysis revealed strong evidence of shared ancestry between *mexicana* and our highland maize samples (*D*, *Z* score > 9.56; Table 1), with *mexicana* introgression estimated by *f*_*d*_ to account for ∼7% of the highland maize genomes.

**Table 1:** Introgression from mexicana to Mexican highland maize and recombination rate by chromosome.

Target	Size (Mb)	*D*	*Z*	*p*-value	Number of events^1^	Total event size (Mb)	Introgression^2^ (%)	Map length (cM)	RR (cM/Mb)
Chr1	301	0.066	6.0	<0.001	84	9.3	3.1	208	0.69
Chr2	238	0.063	5.7	<0.001	67	5.7	2.4	144	0.61
Chr3	232	0.075	5.0	<0.001	85	2	8.8	138	0.60
Chr4	242	0.133	5.4	<0.001	90	24.8	10.2	130	0.54
Chr5	218	0.072	5.5	<0.001	72	8.9	4.1	142	0.65
Chr6	169	0.104	5.2	<0.001	74	11.5	6.8	113	0.67
Chr7	177	0.055	4.5	<0.001	51	5.1	2.9	116	0.66
Chr8	175	0.041	2.6	0.009	61	6.9	4.0	86	0.49
Chr9	157	0.028	1.7	0.09	52	4.7	3.0	100	0.64
Chr10	150	0.051	3.1	0.002	43	4.6	3.0	97	0.65
Total	2,060				679	102	5.0	1,275	0.62

**Notes:**

1 Concatenated 10% outliers.

2 Based on physical size.

**Figure 1:**
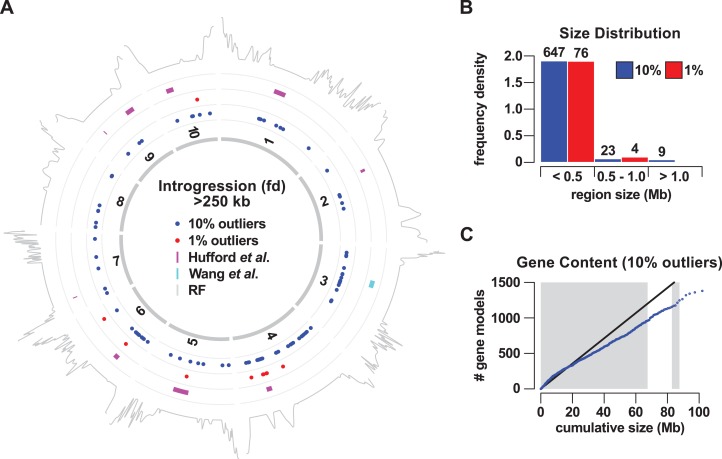
Gene flow from teosinte mexicana to Mexican highland maize. (A) Mapping of the regions of introgression >250 kb in size to the 10 chromosomes of maize (1–10). Colored points indicate the midpoint of regions identified using a 10% (blue) or 1% (red) outlier cut-off. Magenta bars show nine regions previously identified by Hufford et al. (2013). The cyan bar shows a Mb-scale region on chromosome 3 identified by Wang et al. (2017). The gray trace on the outermost track indicates local recombination frequency (log_2_ transformed) estimated from the PT x REV F_2_ population. (B) Size distribution (frequency density) of introgression events using a 10% (blue) or 1% (red) outlier cut-off. Events binned by size <0.5 Mb, 0.5 Mb to <1 Mb (1.0), one Mb or greater. Numbers above bars give the number of events in that class. (C) Cumulative gene count as a function of cumulative physical size of 10% outlier introgression regions, ordered by size. Blue points indicate the cumulative count. The black line shows the expected trend based on total genome size and gene number, under the assumption of a uniform spatial distribution. Alternating gray and white bars indicate size thresholds of the individual events of 0.5, one, 1.5 and >1.5 Mb.

**Figure 2:**
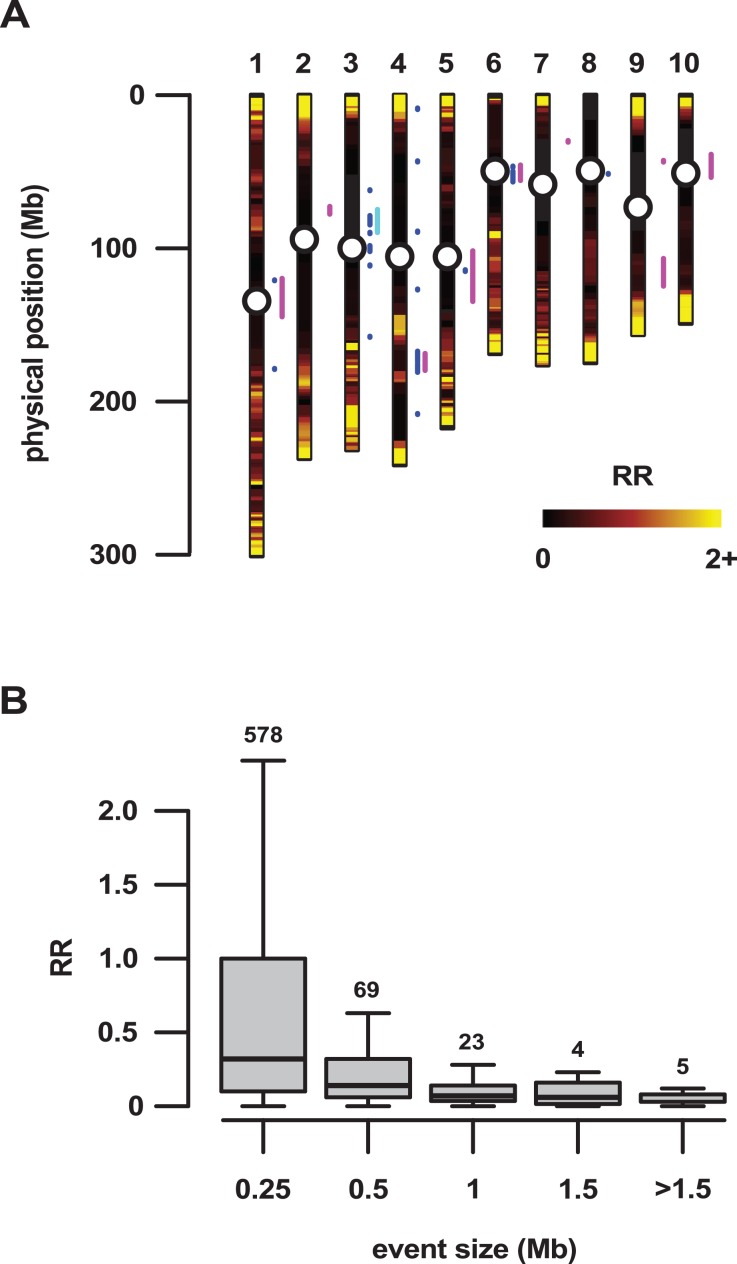
Large regions of introgression are associated with low genetic recombination. (A) Local recombination rate (RR) across the physical space of the 10 maize chromosomes (1–10), estimated from the PT x REV F_2_ population. RR shown from zero to two on a linear black-red-yellow scale. The RR distribution was truncated, with values >2 set to 2 (predominantly high values in the telomeric regions). A total of 32 introgression events >500 kb in size that were identified by concatenation of the top 10% outlying *f_d_* windows are shown as blue line segments to the right of the individual chromosomes. Magenta and cyan line segments show regions previously identified by Hufford et al. (2013) and Wang et al. (2017), respectively. Centromeres shown as open circles. (B) RR as a function of the physical size of introgression events. Events were grouped into size classes, given as the upper threshold on the plot. Boxes show 1st quartile, median and 3rd quartile. Whiskers extend to the most extreme points within 1.5× box length; outlying values beyond this range are not shown. Numbers above the boxes show the number of events in each size class.

To localize introgression within the genome, we calculated *f*_*d*_ in non-overlapping windows of 50 informative sites. We considered relatively high-scoring windows to be positive for introgression, selecting the sets of the top 1% and top 10% outliers for further analysis. Adjacent positive windows were concatenated, defining 80 (8.86 Mb or 0.43% of the genome) and 679 (101.98 Mb or 4.95% of the genome) introgression events for 1% and 10% sets, respectively (Fig. 1; Tables S1 and S2). In light of our genome-wide estimation of 7% *mexicana* ancestry, both the 1% and 10% sets appear to be conservative. Introgression events were distributed across all 10 chromosomes, although not uniformly, with chromosome (chr) 4 particularly enriched (Fig. 1A; Table 1). In the 1% outlier set, 29 of a total of 80 events were located on chr 4, representing 58% of the total introgressed DNA by size. For the 10% outlier set, 90 of 679 events were located on chr 4, representing 24% of the total by size. Genomewide, the size of introgression events in the 1% outlier set ranged from 0.53 to 630 kb. The upper limit was substantially increased in the 10% set, with events ranging from 0.34 to 4,700 kb, indicating that many of the windows between the first and 10th percentile were clustered in the genome. In both 1% and 10% outlier sets, the majority (95% in both cases) of the events were less than 0.5 Mb in size, with these small events also constituting the majority of the total physical introgression size (Fig. 2B). Nine events were identified that were >1 Mb in size, all from the 10% outlier set. These included events that co-localized with previously reported Megabase-scale introgression regions (Fig. 1A; Table S11; Hufford et al., 2013; Romero Navarro et al., 2017; Wang et al., 2017): the *Inv4m* inversion polymorphism on chr 4 (located at 169–180 Mb; represented as 15 closely located events in our analysis), a region on chr 6 (located at 46–57 Mb; four events in our analysis) and a region on chr 3 (located at 75–90 Mb; three events in our analysis).

To assess the potential functional significance of *mexicana* introgression, we examined the gene content of the introgression events on the basis of the B73 reference genome (Table S5). While previously reported Megabase-scale regions harbor a large number of annotated genes, we considered the possibility that smaller regions were largely distributed in gene-poor sections of the genome, perhaps as remnants of historical gene flow, experiencing little purifying selection as a result of limited functionality. Overall, the cumulative distribution of gene number as a function of ordered physical size conformed with the expectation of genome-wide gene density, with a total of 1,380 genes (3.5% of the tested genes) found inside the 10% outlier events (Fig. 1C; Table S5). There was no indication that small introgression events were gene-poor, and it was at the higher end of the size spectrum that gene-density fell slightly below the genome-wide value, consistent with the location of many of the larger events in pericentromeric regions.

### Large introgression events are located in regions of low genetic recombination

**Figure 3:**
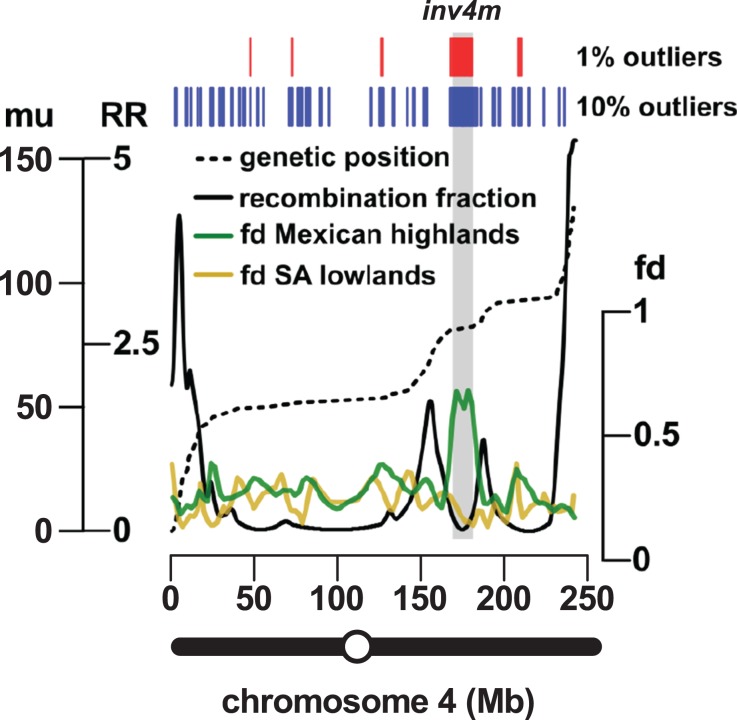
Introgression events on chromosome 4 co-localize with a previously reported inversion. Marey plot (black dashed line) of genetic position in map units (mu; left-hand axis, outer) against physical position in Mb (*x*-axis) across chromosome 4. Local recombination rate (RR; solid black line) was calculated as the derivative of the physical-genetic plot (left-hand axis, inner). Introgression from mexicana to Mexican highland maize was estimated as local *f_d_* (green line; lowess smoothing of sliding window analysis). The result of a similar analysis substituting South American for Mexican highland maize is also shown (brown line). Introgression events identified by selection and concatenation of the top 1% or top 10% outlying windows in the Mexican highland analysis are in red and blue, respectively. The position of the *Inv4m* inversion polymorphism, as previously reported by Hufford et al. (2013), is shown as a gray rectangle. The position of the centromere is indicated as an open circle on the chromosome schematic.

The most reproducible signals of introgression from *mexicana* to Mexican highland maize are associated with Megabase-scale events that co-localize with putative chromosomal inversions (e.g., regions on Chr 3 and Chr 4 reported here and previously by Hufford et al. (2013), Romero Navarro et al. (2017) and Wang et al. (2017)), consistent with the hypothesis that a low local rate of genetic recombination can favor introgression (Kirkpatrick & Barton, 2006). Of course, it is also clear that such large scale events are easier to detect. To characterize the recombination landscape of the PT genome, we generated a genetic linkage map from the cross of PT1 and the lowland Mexican landrace Reventador (RV; partially inbred accession used in the *f*_*d*_ analysis). The total map length was 1,275 cM, with a global RR of 0.61 cM/Mb (based on the size of the B73 v3 physical map). At the level of individual chromosomes, RR ranged from 0.69 on chr 1 to 0.49 on chr 8 (Table 1). As is typical, local RR values were high in the telomeric regions and low around the centromeres (Fig. 2A; Table S6). In addition, we observed variation across the genome with clear recombination hot and cold spots (Fig. 2A). For each introgression event, RR was estimated based on the midpoint location. The RR differed depending on the size of the introgression events (Kruskal–Wallis test, *p* < 0.001; Fig 2B): while small (<250 kb) events were distributed across a range of RR, large regions (>250 kb) were constrained to regions where RR < 0.5 (Fig 2B), with the exception of one event on chr3 (RR = 0.58) and one on chr 4 (RR = 1.91), although, in both cases, RR was reduced compared with their surroundings (Tables S6 and S7). Of the 32 events >0.5 Mb identified from the 10% outlier set, 18 were located in pericentromeric regions (defined as the region for which RR ≤ 0.2 extending from the estimated position of the centromere; Fig. 2A; Table S12), suggesting that we have more large introgression events at pericentromeric regions than expected (χ^2^ = 7.73, d*f* = 1, *p*-value = 0.005), consistent with the idea that highland maize carry centromeric or pericentromeric regions from *mexicana* (Hufford et al., 2013). We compared our map with the maize nested association mapping (NAM) population reference (McMullen et al., 2009), which did not include highland maize material in its construction. We found that the non-pericentromeric regions harboring large (>0.5 Mb) introgression events in our analysis presented lower local rates of recombination than the corresponding positions in the NAM map (paired-sample Wilcoxon test, *p*-value < 0.001; Table S12), suggesting that these regions themselves may be suppressing recombination. The strong signal of introgression across *Inv4m* coincided clearly with a region of low RR, consistent with the segregation of inverted and standard haplotypes in our PT × RV cross (Fig. 3; Hufford et al., 2013; Romero Navarro et al., 2017; Wang et al., 2017).

### Introgression events contribute to the differentiation of Mexican highland and lowland maize

**Figure 4:**
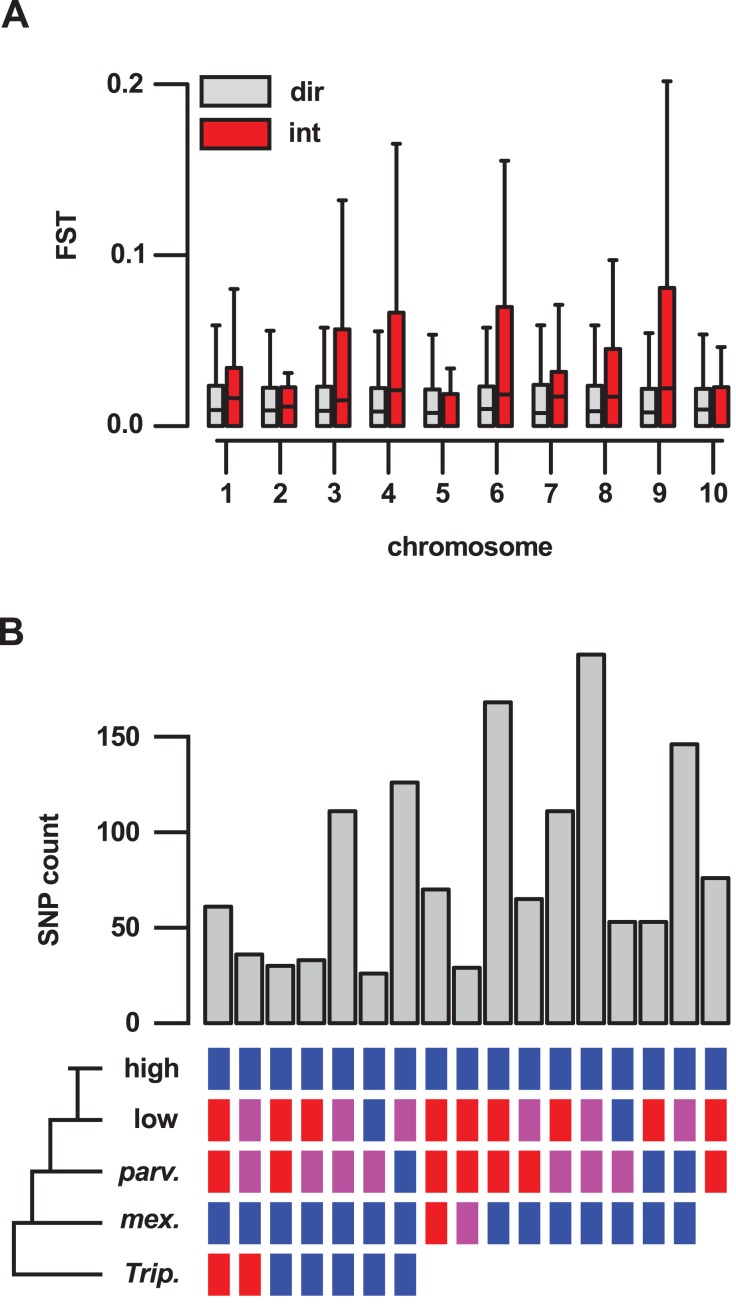
Genes located in introgression regions differentiate highland and lowland Mexican maize and are enriched for high effect SNPs. (A) *F*_*ST*_ between Mexican highland and lowland maize populations, as estimated by Takuno et al. (median *F*_*ST*_ of all SNPs per gene model; 21,029 gene set), for genes located in regions of direct descent (dir) or introgression (int) events, on the 10 chromosomes of maize. Boxes show 1st quartile, median and 3rd quartile. Whiskers extend to the most extreme points within 1.5× box length; outlying values beyond this range are shown as points, including a number of extreme outliers in both direct and introgression regions. (B) The number of high or moderate effect SNPs fixed in the three Mexican highland maize individuals with respect to their distribution in the other samples. Colored boxes indicate SNPs fixed for the alternate allele (blue), the reference allele (red) or segregating (magenta) in a given group. Blanks indicate that SNPs could be not called. Only sets containing greater than 25 SNPs are shown.

To better understand the importance of introgression in the differentiation of highland and lowland Mexican maize, we examined a previously published *F_ST_* data set (Takuno et al., 2015), comparing genes inside and outside of our introgression events (Table S5). For 409 genes reported to show significant differentiation (from a total of 21,029 genes for which an *F_ST_* estimate was available), 62 (15%) were located in the set of 10% outlier introgression events, an enrichment over the genome-wide expectation (χ^2^ = 205, d*f* = 1, *p*-value < 0.001; Table S13). This trend was driven, in part, by the large number of high *F_ST_* genes within introgression events on chr 4 (34 of the total of 62 high *F_ST_* genes within introgression), although the enrichment remained even after removal of chr 4 from the data set (χ^2^ = 57, d*f* = 1, *p*-value < 0.001; Table S14). Across the 21,029 genes for which an estimate was available, the median *F_ST_* value was significantly higher for 636 genes located in introgression regions than for the remaining 20,393 genes (Wilcox test, *p*-value < 0.001). When chromosomes were considered individually, chr 3, 4, 8 and 9 showed a significant (*p* < 0.01) difference in *F_ST_* (Fig. 4A).

### Introgression events contain variation of potential significance for protein function

To assess functional variation, we categorized the SNPs identified in genes on the basis of their possible impact on encoded proteins using SnpEff (Cingolani et al., 2012). Genes located in introgression events showed an excess of high (χ^2^ = 35.03, d*f* = 1, *p*-value < 0.001; Table S15) and moderate (χ^2^ = 222.72, d*f* = 1, *p*-value < 0.001; Table S16) effects fixed in our sample of three genomes (six alleles). In total, 40 of the 1,380 genes in introgression events were homozygous for the alternate allele at one of more high-effect SNPs across all three highland maize individuals (45 SNPs in total; Table S8). An additional 502 genes were fixed for the alternate allele at one or more moderate-effect SNPs (1,740 SNPs in total; Table S8). We further categorized fixed high- and moderate-effect SNPs with respect to the other samples in our analysis (Fig. 4B; Table S8). The majority of SNPs in introgression regions fixed for the alternate allele in our highland maize samples were also fixed for the alternate allele in *mexicana* (1,346 of 1,785 SNPs). Of these, 61 highland-fixed SNPs (located in 40 genes) unambiguously followed the “ABBA” pattern used initially for the selection of introgression regions (i.e., fixed for the alternate allele in highland maize and *mexicana*; fixed for the reference allele in *Tripsacum*, lowland maize, and, although not included in the earlier analysis, also *parviglumis*). A further 168 highland-fixed SNPs (located in 96 genes) were not called in *Tripcascum* (and therefore, were not used in our estimation *f*_*d*_), but were fixed for the alternate allele in highland and *mexicana* samples and fixed for the reference allele in lowland and *parviglumis* samples, consistent with introgression. The largest category resulting from this grouping consisted of 193 SNPs (located in 104 genes) that were not called in *Tripsacum*, were fixed alternate in highland and *mexicana* samples, and segregating in lowland and *parviglumis* samples. Such SNPs differentiate highland and lowland individuals in both teosinte and maize, and their distribution within putative introgression events is consistent with an introgressed origin. A total of 70 highland-fixed SNPs (located in 41 genes), including 48 in the *Inv4m* region, were private to our highland genomes.

To combine variant effect prediction with annotated gene function, we cross referenced the list of genes in introgression events with the classical maize gene list, a curated set of 4,908 well characterized genes (the “combined set” gene list was obtained from www.maizegdb.org/gene_center/gene and filtered for unique gene identifiers). Considering the classical genes located in introgression regions as a whole, a diverse range of functions are represented, many that are potentially significant to morphology or environmental responses (Table 2). Intriguing examples include the *Bx8* gene required for the biosynthesis of benzoxazinoid defense compounds (Frey et al., 1997), the phosphorus homeostasis gene *Pho1;2a* (Salazar-Vidal et al., 2016), the flowering-time locus *Gi2* (Mendoza et al., 2012), and various genes related to phytohormone biosynthesis, ear morphology and grain development (Table 2). These last include the genes *Compact Plant2* (*Ct2*), *Fasciated Ear3* (*Fea3*) and *Tunicate* (*Tu1*) that play a role in the regulation of plant meristems (Han, Jackson & Martienssen, 2012; Je et al., 2016; Wu et al., 2018; Han, Jackson & Martienssen, 2012), the genes *Nana Plant1* (*Na1*) and *Nana2-like1* (*Natl1*) involved in brassinosteroid biosynthesis (Hartwig et al., 2011; Best et al., 2016), *Aminocyclopropane carboxylate oxidase20* (*Acco20*) involved in ethylene biosynthesis (Mira, Hill & Stasolla, 2016), and the genes *Dwarf8* (*D8*) and *Kaurene oxidase1* (*Ko1*) that play a role in gibberellic acid signaling (Peng et al., 1999).

**Table 2:** Selected classic genes located in introgression events.

Gene	ID	Function	Chr	Pos (Mb)	Top 10%	Top 1%
*A1*	GRMZM2G026930	Pigments	3	216	X	
*Acco20*	GRMZM2G126732	Hormones	4	178	X	X
*Bx8*	GRMZM2G085054	Defense	4	3	X	
*Cle24*	GRMZM2G123818	Morphology	4	170	X	
*Ct2*	GRMZM2G064732	Morphology	1	16	X	
*D8*	GRMZM2G144744	Hormones	1	266	X	
*Fea3*	GRMZM2G166524	Morphology	3	291	X	
*Fl3*	GRMZM2G006585	Grain development	8	52	X	
*Gi2*	GRMZM5G844173	Flowering	3	9	X	
*Ko1*	GRMZM2G059308	Hormones	9	80	X	
*Na1*	GRMZM2G449033	Hormones	3	179	X	
*Natl1*	GRMZM2G455658	Hormones	4	169	X	X
*O1*	GRMZM2G449909	Grain development	4	177	X	
*Orp2*	GRMZM2G005024	Grain development	10	84	X	
*Sbe1*	GRMZM2G088753	Grain development	5	63	X	
*Ss5*	GRMZM2G130043	Grain development	4	173	X	
*Su4*	GRMZM2G090905	Grain development	6	145	X	
*Pho1;2a*	GRMZM2G466545	Nutrition	4	172	X	X
*Tu1*	GRMZM2G370777	Morphology	4	179	X	X

To look at the possible implication of post-domestication gene flow during the early development of cultivated maize, introgression events were compared with the location of previously reported domestication and improvement genes (Hufford et al., 2012b). Of 420 reported domestication candidates present in the B73 v3 reference genome annotation (i.e., genes showing a reduction in diversity and increased differentiation between teosinte and landrace maize), 17 (3.6%) were located in introgression events (based on 10% outliers). Similarly, of 529 annotated improvement candidates (i.e., genes showing a reduction in diversity between landrace maize and modern inbred lines), 22 (3.8%) were located in introgression regions. For both domestication and improvement candidates, the proportion within introgression events mirrored the genome-wide value of 3.5% (domestication: χ^2^ = 0.2185, d*f* = 1, *p*-value = 0.64; improvement: χ^2^ = 0.48451, d*f* = 1, *p*-value = 0.48). As such, we see no evidence that these candidates are refractory to introgression.

### Introgression events on chromosome 9 co-localize with a previously-reported QTL for sheath pubescence

**Figure 5:**
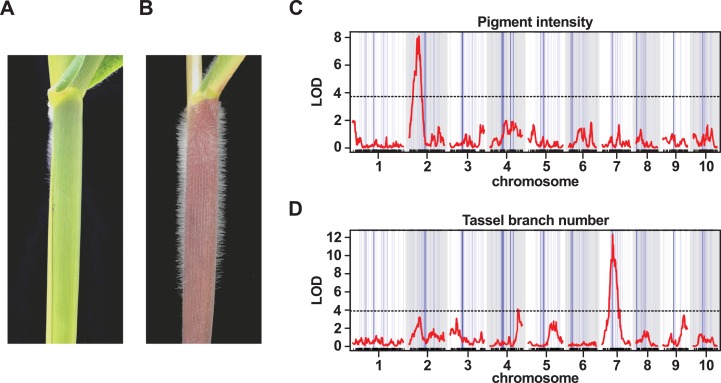
QTL peaks associated with morphological traits do not co-localize with large-scale introgression events. (A) The lowland landrace Reventador (RV) exhibits the non-pigmented, glabrous stem, typical of lowland maize, in contrast to (B) Palomero Toluqueño (PT) and other Mexican highland maize landraces which present extensive stem pigmentation and pubescence. Whole-genome QTL scans for the intensity of (C) stem pigmentation (pigment intensity) and (D) the degree of branching of the male inflorescence (tassel branch number). The horizontal axis shows genetic position across the ten maize chromosomes in the PT × RV map, tick-marks indicating marker position, alternating white/gray banding in the plot area indicating chromosomes. Vertical blue lines across the plot area indicate the estimated position of introgression events on the genetic map, line width proportional to event size. The vertical axis shows LOD support for the presence of a QTL. The red trace shows the output from a single-QTL interval scan. The horizontal broken black-lines indicate the 5% LOD threshold for each trait, as determined by permutation. Major peaks for pigment intensity and tassel branch number are located on chromosomes 2 and 7, respectively.

One of the most striking morphological characteristics of Mexican highland maize is the presence of pronounced stem pubescence (Figs. 5A and 5B; Wellhausen et al., 1952). In a previous study, evidence of *mexicana* introgression was identified on chr 9 (106.5125.5 Mb; Hufford et al., 2013), co-localizing with *macrohairless1* (*mhl1*), a locus linked with production of macrohairs on the adaxial surface of the leaf blade in inbred maize lines (∼115 Mb; Moose, Lauter & Carlson, 2004). We recovered a single introgression event in this region of chr 9 in our 1% outlier set, and a number of events in our 10% outlier set (Tables S1 and S2). A previous experiment to map sheath pubescence in a cross between *parviglumis* and *mexicana* identified a major effect QTL on the long-arm of chr 9, consistent with the action of *mexicana*-specific neomorphic allele of *mhl1* extending the production of macrohairs from leaf blade to sheath (Lauter et al., 2004). In a further study, using recombinant inbred lines derived from the cross between B73 and PT, there was also evidence to link a QTL in the *mhl1* region to stem pubescence (Aguilar-Rangel, 2018). Here, we attempted to map stem pubescence in the F_2_ progeny of our PT × RV cross. Upon evaluation, however, we found that the majority of the F_2_ plants (140 of 157) presented stem macrohairs (scored on a semi-quantitative scale). While this provided insufficient variation for successful QTL mapping, it may indicate the action of multiple dominant-acting factors. We confirmed that the lowland RV parent did not show stem pubescence. We anticipate that the use of inbred material to reduce the confounding effects of dominance, along with fine-scaled quantitative evaluation, would provide a better characterization of the genetic architecture of stem macrohair production in the PT × RV cross.

### QTL peaks associated with morphological and flowering traits do not co-localize with introgression events

In addition to stem pubescence, we evaluated the PT × RV F_2_ population for a number of further morphological and flowering time traits to explore any possible association with introgression. QTL were identified associated with stem pigment intensity (INT), stem pigment extent (EXT), tassel (male inflorescence) branch number (TBN), tassel length (TL) and days to anthesis (male flowering; DTA) (Table 3). The QTL intervals themselves were large (typically, tens of Mbs), and, necessarily, contained multiple introgression events. In no instance, however, did the marker closest to a QTL peak fall directly within one of our introgression events (Fig. 5B; Table 3). Given the limited resolution of our mapping, we looked for instances where we might identify a candidate gene within a given QTL interval for the purpose of evaluating local introgression; two such candidates are discussed below.

**Table 3:** QTL associated with plant morphology and flowering time.

QTL	Chr	Peak(Mb)	95% Interval (Mb)	Var (%)	LOD	Candidate gene
qINT-2	2	19	12–21	23	9.2	*B1*
qPAT-2	2	13	12–20	18	6.7	*B1*
qTBN-2	2	18	3–196	8	4.0	
qTBN-7	7	98	37–118	31	13.2	*Ra1*
qTL-4	4	30	10–155	10	3.8	
qTL-5	5	2	1–3	12	4.5	
qDTA-8	8	117	1–125	14	4.5	*Zcn8*

The qTBN-7 interval contains the candidate gene *Ramosa1* (*Ra1*. GRMZM2G003927. Chr 7: 110 Mb). The *Ra1* product has been characterized to restrict production of long-branches in both the male and female inflorescences (Vollbrecht et al., 2005). When we examined the window containing *Ra1* in our analysis, we found no evidence of introgression (*f*_*d*_ = 0.20). This is perhaps not too surprising given that *mexicana*, although described to present lower TBN than *parviglumis* (Doebley, 1983), does not present the extreme reduction in tassel branching that is characteristic of Mexican highland maize.

The stem pigment QTL qINT-2 and qPAT-2 overlap on chr 2, defining an interval that contains the candidate gene *B1* (GRMZM2G172795. Chr 2: 19 Mb). The *B1* gene encodes a basic helix-loop-helix transcription factor that regulates the tissue-specific biosynthesis of anthocyanins (Goff, Cone & Chandler, 1992; Sharma et al., 2011). In contrast, to greatly reduce tassel branching, stem pigmentation is a trait shared by Mexican highland maize and *mexicana*. Furthermore, there is evidence that allelic variation at *B1* is linked to stem pigmentation differences between *parviglumis* and *mexicana* (Selinger & Chandler, 1999; Lauter et al., 2004). Nonetheless, inspection of the window containing *B1* in our analysis found no evidence of introgression (*f_d_* = 0.22), indicating that although variation at *B1* may drive stem pigmentation in both *mexicana* and Mexican highland maize, the underlying alleles may have independent origins.

## Discussion

Study of a number of crops has begun to indicate the significance of post-domestication gene flow during the diversification and spread of cultivated varieties (Hufford et al., 2013; Poets et al., 2015; Bredeson et al., 2016; Rendón-Anaya et al., 2017). In line with previous reports (Hufford et al., 2013; Wang et al., 2017), we have detected significant genome-wide introgression from *mexicana* to Mexican highland maize. We estimated genome-wide introgression at ∼7%, and we mapped specific introgression events constituting ∼5% of the total physical space. Our values are somewhat lower than other estimates, that have ranged up to 20% (Matsuoka et al., 2002; Hufford et al., 2013; Wang et al., 2017), reflecting the conservative criteria we employed, and, potentially, the result of differences among the individuals in our sampling. Specifically, the differing sources of PT and MM samples might be reflected in distinct patterns of introgression. Similarly, we do not distinguish between introgression proceeding or contemporary with the early diversification of the Mexican highland landrace group, and later events that might be restricted to specific populations or races. Our analysis will largely have identified events that are shared between PT and MM samples, consistent with both high frequency in contemporary populations, and an origin early in the development of the Mexican highland group.

Previous reports of *mexicana* introgression to Mexican highland maize have focused on Mb-scale regions, events also recovered in our analysis. In addition, we also identified large numbers of small (<500 kb) events, that, collectively, constituted the bulk of the total introgression. Introgression events presented a gene-density equivalent to the genome as a whole, contrary to the hypothesis that they were harbored in gene-poor regions, possibly remnants of historical gene-flow with little functional significance. Nonetheless, 262 events (∼10% of the total introgression by size), with an average size of ∼37 kb, did not contain any annotated genes. A further 162 events contained only a single annotated gene. Small, single gene events, presumably the result of recombination following historical hybridization, would not be impacted by the negative effects of linked deleterious alleles, nor would they participate in hitchhiking through linkage to beneficial variants (Barton & Bengtsson, 1986; Kirkpatrick & Barton, 2006). As such, the persistence of single-gene introgression events would be predicted to reflect directly the fitness effects of associated allelic variants. Across all introgression events, we identified a number of genes that have previously been demonstrated to play major roles in maize development and growth, including well-characterized hormone-signaling genes. Variation at loci related to phytohormone signaling has the potential to trigger pleiotropic effects, impacting, for example, flowering time, morphology and stress tolerance. Indeed, the capacity to retune simultaneously multiple aspects of plant morphology, phenology and stress biology make hormone pathways compelling candidates as drivers of a collective adaptive syndrome. Gene-level analyses were conducted on the basis of the B73 genome annotation. Given the extensive copy-number variation known to be present in maize, it will be interesting to re-evaluate introgression gene content when further genome assemblies become available for landrace maize and maize wild-relatives.

Among the genes identified to be in introgression events, we recovered previously characterized domestication and improvement candidates, at a rate equivalent to that observed genome-wide. This observation somewhat contradicts an earlier report finding that regions of introgression from *mexicana* to maize harbored fewer domestication candidates, while regions resistant to such introgression were enriched for domestication candidates (Hufford et al., 2013). It may be significant that we employed a greater number of markers than were used in the previous report, and identified a larger number of small events. All the domestication candidates we identified in introgression regions were in events <775 kb in size (although four candidates were located in small events co-localizing with the *Inv4m* region).

Although many SNPs with potential functional relevance were identified in well-supported gene models, we were unable to link introgression to the phenotypic traits we evaluated. In the case of stem pubescence, we did not observe sufficient variation to permit QTL mapping. Nonetheless, we did identify introgression events in the *mhl1* region of chr 9, that has been previously linked with stem pubescence in both *mexicana* and Mexican highland maize in other studies (Lauter et al., 2004; Aguilar-Rangel, 2018). For stem pigmentation and tassel branch number, we mapped large-effect QTL that co-localized with high-confidence candidate genes. The *Ra1* candidate has also been linked to tassel branch number variation in a maize × *parviglumis* population, the *parviglumis* allele increasing branching threefold with respect to the maize allele (Xu et al., 2017). PT is characterized by greatly reduced tassel branching (often the tassel is a single, unbranched spike), consistent with a gain of *Ra1* function with respect to typical maize varieties, and reminiscent of the phenotype seen in maize *liguleless* and *unbranched* mutants (Wellhausen et al., 1952; Walsh & Freeling, 1999; Chuck et al., 2014). As such, we can hypothesize an allelic series of increasing *Ra1* function from *parviglumis*, through lowland maize, to Mexican highland maize. With respect to stem pubescence, a number of functional variants of *B1* have been described, and differences in stem pigmentation linked to transposon insertion in the region upstream of *B1* (Radicella et al., 1992; Selinger & Chandler, 1999, 2001). Significantly, two previously characterized *B1* alleles from *mexicana* were reported to present a different upstream structure to an allele from the Mexican highland landrace Cacahuacintle (Selinger & Chandler, 1999). Although the resolution of our mapping limited broader conclusions concerning the impact of introgression, in *Ra1* and *B1* we have identified compelling candidates linked to large-effects in two of the most characteristic morphological traits of Mexican highland maize. The fact that we see no evidence for introgression at either *Ra1* or *B1* may suggest that founder populations in the Mexican highlands contained sufficient standing genetic diversity for these characteristic traits to arise without recourse to gene flow. It is interesting to note that stem pigmentation is also prevalent in the demographically distinct maize races of highland South America, where introgression from *mexicana* is considered to be absent (Wellhausen, 1957; Wang et al., 2017).

Notwithstanding the results of our QTL analysis, the extent of *mexicana* introgression, and the number of variants identified, argues for a functional impact. The *Inv4m* region has previously been linked to flowering time in a large-scale association analysis (Romero Navarro et al., 2017). The apparent discrepancy with respect to our observations may reflect the fact that the evaluation was carried out in a lowland environment, or result from epistatic interactions that we were not capable of detecting in our experiment. Indeed, it may well be that the broader phenotypic effects associated with introgression are conditional on growth at high elevation, acting in the modification of major QTL, in responses to biotic and abiotic stress, and in subtle, but significant, contributions to yield and harvest quality traits.

## Conclusion

We detected significant genome-wide introgression from *mexicana* to Mexican highland maize. Employing conservative criteria, we mapped specific introgression events within the genome, constituting ∼5% of the total physical space. Introgression events presented a gene-density equivalent to the genome as a whole, and contained a significant number of genes that have previously been demonstrated to play major roles in maize development and growth. Although potential functional variants were identified, we were unable to link introgression to phenotypic traits. While it is possible that that founder populations in the Mexican highlands contained sufficient standing genetic diversity to support the adoption of the basic morphology and phenology characteristic of modern highland varieties, the extent of *mexicana* introgression, and the number of variants identified, nonetheless argues for a functional impact. We suggest that this impact might yet be identified in modification of major QTL, in responses to biotic and abiotic stress, and in contributions to yield and harvest quality traits under highland conditions.

## Supplemental Information

10.7717/peerj.6815/supp-1 Supplemental Information 1 Figure S1 and Tables S10–S16. Figure S1. Mexican highland maize is cultivated sympatrically with teosinte mexicana. Niche suitability map for teosintes parviglumis and mexicana, and the highland maize landrace Palomero Toluqueño. Supplemental tables: Table S10. Genome sequence data generated in this study; Table S11. Introgression events larger than one Mb identified from the top 10% outliers; Table S12. Introgression events larger than 0.5 Mb located outside pericentromeric regions; Table S13. Contingency table for 21,029 genes with *F_ST_* estimates; Table S14. Contingency table for 18,855 genes with *F_ST_* estimates outside chr 4; Table S15. Contingency table for genes with fixed high effect SNPs; Table S16. Contingency table for genes with fixed moderate effect SNPs.

10.7717/peerj.6815/supp-2 Supplemental Information 2 Top 1% *f*_*d*_ outlier windows. Introgression events identified by concatenation of the top 1% scoring windows from the genomewide *f*_*d*_ scan

10.7717/peerj.6815/supp-3 Supplemental Information 3 Top 10% *f*_*d*_ outlier windows. Introgression events identified by concatenation of the top 10% scoring windows from the genomewide *f*_*d*_ scan

10.7717/peerj.6815/supp-4 Supplemental Information 4 *f*_*d*_ output by windows—highland test set. Raw output from *f_d_* analysis. Mexican highland maize in test position.

10.7717/peerj.6815/supp-5 Supplemental Information 5 *f*_*d*_ output by windows—lowland null set. Raw output from *f_d_* analysis. South American maize in test position.

10.7717/peerj.6815/supp-6 Supplemental Information 6 Introgression and recombination at gene models. Maize B73 ref_gen v3 gene models including annotation, indicating presence in introgression regions, local recombination rate, associated SNPs, SNP effect and Mexican highland-lowland *F_ST_*.

10.7717/peerj.6815/supp-7 Supplemental Information 7 Local recombination rate at grid positions. Local recombination rate in the RV × PT cross, estimated every 500 kb.

10.7717/peerj.6815/supp-8 Supplemental Information 8 Local recombination rate at markers. Local recombination rate in the RV × PT cross, estimated at every marker.

10.7717/peerj.6815/supp-9 Supplemental Information 9 SNPs at introgression regions fixed in Mexican highland individuals. Table of high and moderate effect SNPs fixed in Mexican highland samples.

10.7717/peerj.6815/supp-10 Supplemental Information 10 R/QTL cross object for RV × PT cross. R/QTL cross object containing phenotypic and marker data for the RV × PT cross.

## Additional Information and Declarations

### DNA Deposition

The following information was supplied regarding the deposition of DNA sequences: Genome sequence data is available from National Center for Biotechnology Information (NCBI) Sequence Read Archive (SRA) database (PRJNA511379).

### Data Availability

The following information was supplied regarding data availability: Novel scripts are available at GitHub: https://github.com/ericgonzalezs/Characterization_of_introgression_from_Zea_mays_ssp._mexicana_to_Mexican_highland_maize.
