# Supplementary material for: Characterization of introgression from the teosinte Zea mays ssp. mexicana to Mexican highland maize
Source: PeerJ. 2019 May 3;7:e6815. doi: 10.7717/peerj.6815 (PMC6501764; doi:10.7717/peerj.6815)
Supplement: Supplemental Information 1 — Figure S1. Mexican highland maize is cultivated sympatrically with teosinte mexicana. Niche suitability map for teosintes parviglumis and mexicana, and the highland maize landrace Palomero Toluqueño. Supplemental tables: Table S10. Genome sequence data generated in this study; Table S11. Introgression events larger than one Mb identified from the top 10% outliers; Table S12. Introgression events larger than 0.5 Mb located outside pericentromeric regions; Table S13. Contingency table for 21,029 genes with FST estimates; Table S14. Contingency table for 18,855 genes with FST estimates outside chr 4; Table S15. Contingency table for genes with fixed high effect SNPs; Table S16. Contingency table for genes with fixed moderate effect SNPs. [file peerj-07-6815-s001.pdf]

Gonzalez-Segovia et al. Supplemental Materials

List of Supplemental Tables provided in additional files

Table S1. Introgression events from the Top 1% outlying windows.

Table S2 Introgression events from the Top 10% outlying windows.

Table S3. Raw Fd output by window for highland test data set

Table S4. Raw Fd output by window South American lowland null data set

Table S5. Table of maize genes indicating their presence in introgression regions, local recombination rate, associated SNPs, SNP effect and Mexican highland-lowland Fst.

Table S6. Local recombination rate in the RV x PT cross, estimated every 500kb

Table S7. Local recombination rate in the RV x PT cross, estimated at every marker

Table S8. Table of high and moderate effect SNPs fixed in Mexican highland samples

Table S9. R/QTL cross object containing phenotypic and marker data for the RV x PT cross

Other Supplemental Tables (S10 - S16)

Table S10. Genome sequence data generated in this study

Identifier	Accession	Elevation (m)	Source	Coverage	Filtered SNPs
PT1	MEXI5	2, 597	CIMMyT	41x	63,802,556
PT2	MEXI5	2, 597	CIMMyT	42x	64,028,598
MM1	TC313	2, 271	Carrera	70x	66,586,613

Table S11. Introgression events larger than 1 Mb identified from the top 10% outliers

Chromosome	Start (Mb)	End (Mb)	Size (Mb)	Gene count	RR (cM/Mb)
3	80.3	85.0	4.7	21	0.0
3	98.6	100.9	2.3	22	0.03
4	167.5	168.6	1.1	6	0.23
4	168.8	171.0	2.2	55	0.12
4	172.5	173.8	1.3	38	0.03
4	177.1	178.4	1.3	33	0.09
5	114.3	115.4	1.1	13	0.00
6	50.0	53.4	3.3	18	0.08
6	54.0	55.8	1.9	18	0.08

Table S12. Introgression events larger than 0.5 Mb located outside pericentromeric regions

chr	start	end	size	RR (cM/Mb)	RR NAM(cM/Mb)
1	178776090	179369070	592980	0.33	0.53
3	157784932	158395423	610491	0.58	0.81
4	8777068	9697246	920178	1.91	2.62
4	167471523	168578399	1106876	0.23	0.78
4	168782414	170998801	2216387	0.12	0.83
4	171586337	172138580	552243	0.05	1.08
4	172473846	173742820	1268974	0.03	1.2
4	173839003	174497224	658221	0.02	1.49
4	175801991	176531617	729626	0.04	1.61
4	177083713	178412600	1328887	0.09	1.53
4	178697931	179386468	688537	0.15	1.35

4	180085104	180656401	571297	0.23	1.22
4	180683844	181252546	568702	0.28	1.22
4	208214069	208771311	557242	0.02	0.16

Table S13. Contingency table for 21, 029 genes with Fst estimates

	In introgression	Not in introgression
Sig Fst	62	574
Non sig Fst	347	20, 046

Table S14. Contingency table for 18, 855 genes with Fst estimates outside chr 4

	In introgression	Not in introgression
Sig Fst	28	311
Non sig Fst	383	18, 133

Table S15. Contingency table for genes with fixed high effect SNPs

	In introgression	Not in introgression
High effects	40	422
Other SNPs	1, 340	37, 503

Table S16. Contingency table for genes with fixed moderate effect SNPs

	In introgression	Not in introgression
Moderate effects	502	7, 528
Other SNPs	878	30, 397

Supplemental Figures

Figure S1. Mexican highland maize is cultivated sympatrically with teosinte *mexicana*. Ecological niche suitability map for teosintes mexicana and parviglumis, and the highland maize landrace Palomero Toluqueño. Regions of distribution overlap between Palomero Toluqueño and mexicana or parviglumis are indicated in yellow and light blue, respectively. The collection locations of the highland accessions PT and MM are shown. Mexico City is marked for reference.

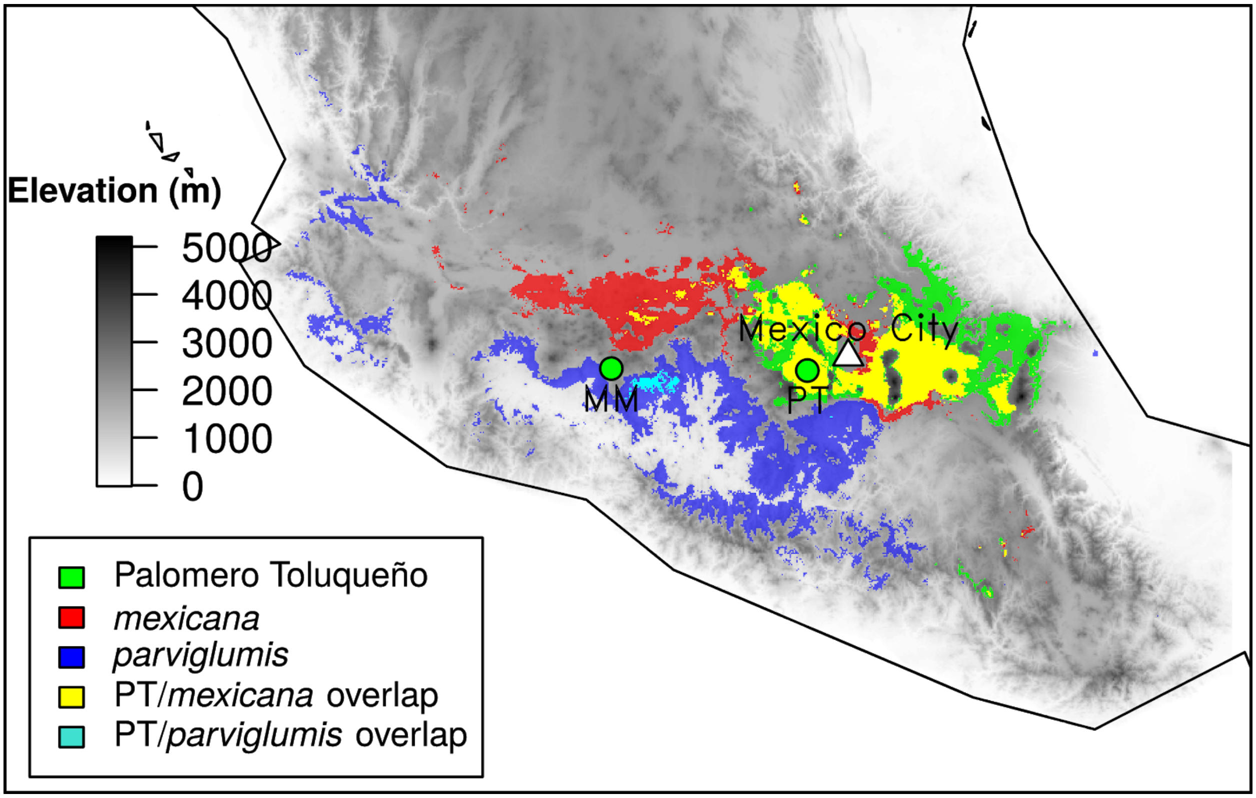

Fick SE, Hijmans RJ (2017). WorldClim 2: new 1-km spatial resolution climate surfaces for global land areas. International journal of climatology, 37(12), 4302-4315.

GADM. (2011). GADM database of Global Administrative Areas v 2.0.

Hijmans RJ, Elith J (2013). Species distribution modeling with R. R CRAN Project.

Hijmans RJ, van Etten J (2012). raster: Geographic analysis and modeling with raster data. R package version 2.0–12.

Hijmans RJ, Phillips S, Leathwick J, Elith J, Hijmans MRJ (2017). Package 'dismo'. Circles, 9(1), 1-68.

Lewin-Koh NJ, Bivand R, Pebesma J, Archer E, Baddeley A, Giraudoux DG, ... & Sebastian P (2012). Package ‘maptools’. Internet: http://cran. r-project. org/web/packages/maptools/maptools. pdf (30.1. 2012).

Phillips SJ, Anderson RP, Schapire RE (2006). Maximum entropy modeling of species geographic distributions. Ecological modelling, 190(3-4), 231-259.
